# Scanning Electron Microscopy of Male Terminalia and Its Application to Species Recognition and Phylogenetic Reconstruction in the *Drosophila saltans* Group

**DOI:** 10.1371/journal.pone.0097156

**Published:** 2014-06-10

**Authors:** Tiago Alves Jorge Souza, Fernando Barbosa Noll, Hermione Elly Melara de Campos Bicudo, Lilian Madi-Ravazzi

**Affiliations:** 1 Department of Genetics, São Paulo University, USP, Ribeirão Preto, Brazil; 2 Department of Zoology and Botany, São Paulo State University, UNESP/IBILCE, São José do Rio Preto, São Paulo, Brazil; 3 Department of Biology, São Paulo State University, UNESP/IBILCE, São José do Rio Preto, São Paulo, Brazil; 4 Department of Biology, São Paulo State University, UNESP/IBILCE, São José do Rio Preto, São Paulo, Brazil; University of Arkansas, United States of America

## Abstract

The *Drosophila saltans* group consists of five subgroups and 21 species, most of which have been identified only by morphological aspects of the male terminalia revealed by drawings using a camera lucida and a bright-field microscope. However, several species in the group, mainly those included in the *saltans* subgroup, are difficult to differentiate using only these characteristics. In this study, we used scanning electron microscopy (SEM) to analyze 19 structures of the male terminalia in 10 species from the five *saltans* subgroups. Among these structures, nine could be identified only through SEM analysis. We aimed to find other characteristics useful for morphological recognition of these species and to use these characteristics for phylogenetic reconstruction. These morphological differences enabled us to effectively distinguish among sibling species. These findings confirmed the monophyly of this group as previously determined in evolutionary studies based on other markers. The single most parsimonious tree (CI = 87 and RI = 90) indicated that the *cordata* subgroup is the most basal lineage and the *saltans* subgroup is the most apical lineage, as shown in earlier studies based on morphological data. However, our findings differed somewhat from these studies with respect to the phylogenetic relationships of species in the *saltans* group indicating that this group is still a puzzle that remains to be deciphered.

## Introduction

The *saltans* group of *Drosophila* (Diptera, Drosophilidae) is divided into five subgroups on the basis of morphological features [1], [2]. Two of these subgroups (*parasaltans* and *cordata*) are found in the Neotropics; the remaining subgroups (*elliptica*, *sturtevanti* and *saltans*) are found in both the Neotropics and the Nearctic regions [2].

In addition to these morphological features, several other characteristics of the group have been studied with a focus on the phylogenetic relationships of the species and subgroups. The markers used in these evolutionary studies include morphological characters [3], [4], chromosomal polymorphisms [5], esterase patterns [6], [7], [8], degree of reproductive isolation [5], [9], gene sequence variation [10], [11] and transposable elements [12], [13], [14], [15], [16].

Most of the phylogenetic relationships presented in these earlier studies differ from each other. Molecular analyses using the *COI*, *COII*, *ITS1*, *Adh* and *Xdh* genes were expected to be highly informative, but provided different topologies among subgroups and even among species [17]. However, they confirmed the monophyly of the group, which had previously been demonstrated using other markers [3]–[16].

Within the *saltans* group, sibling species have been differentiated almost exclusively via analysis of the male terminalia. This structure is considered one of the more variable and rapidly evolving morphological traits in several groups of organisms that exhibit internal fertilization [18], and it has been shown to have considerable taxonomic value for several groups of organisms [19]. The most informative studies on the morphology and taxonomy of the *saltans* group that are available in the literature were performed by Magalhães and Björnberg [1] and Magalhães [2], who differentiated the species into the five subgroups. In these studies, the structures of the male terminalia were represented in drawings obtained using a camera lucida. However, because the structures of the male terminalia of this group are complex, rich in detail, and similar among species from the same subgroup, identifying species by comparing newly collected specimens with the available terminalia diagrams remains difficult.

The inclusion of SEM in analyses of male terminalia has improved the recognition of different strains, species and subfamilies of several different species (e.g., the screwworms *Cochlioma homnivorax* and *C. macellaria* [20] and the Aphididae family of insects [21]). This methodology has proved to be efficient in studies that integrate morphology and molecular data for phylogenetic purposes, as in the case of the invasive drosophilid *Zaprionus indianus* [22].

In order to better resolve species diagnostics and phylogenetic relationships, we analysed nineteen male terminalia traits using SEM for species in the *saltans* group. In this paper, we also compared the results of our phylogenetic analysis to other published studies in order to evaluate the potential advantages of using aedeagus fine structure compared to other sources of data.

## Materials and Methods

### Species and strains

The species and strains examined in this study are listed in Table 1. Cultures were reared on a banana-agar-medium and housed in the biological laboratory of UNESP/IBILCE at 20°C±1°C.

**Table 1:** Species and strains analysed in this study with geographic origins and collectors/identifiers and date of the collections.

Subgroups	Species	Strains	Geographic Origin	Collectors/Identifiers	Date
*saltans*	*D. prosaltans*	***PBB	Barra Bonita/SP/Brazil	Lopes, F.R	1999
	*D. prosaltans*	***PSR	Santana do Riacho/MG/Brazil	Vilela, C.R.	1995
	*D. lusaltans*	**B44 (14045-0891.00)	Petionville/Haiti	Reed, W.	1959
	*D. saltans*	**SAM (14045-0911.00)	San José/Costa Rica	Wasserman, M.	1956
	*D. austrosaltans*	***TUR	Turmalina/SP/Brazil	Penariol, L./Madi-Ravazzi, L.	2008
*sturtevanti*	*D. sturtevanti*	***TAQ	Taquaritinga/SP/Brazil	Penariol, L./Madi-Ravazzi, L.	2008
	*D. sturtevanti*	***TUR	Turmalina/SP/Brazil	Penariol, L./Madi-Ravazzi, L.	2008
	*D. dacunhai*	*JD (28.990.28.999)	Kingston/Jamaica	Mourão; Bicudo	
	*D. milleri*	**EY (14043-0861.00)	El Yunque/Puerto Rico	William Reed	1956
*parasaltans*	*D. parasaltans*	***B17-5	Belém/PA/Brazil	Magalhães, E.	1973
*cordata*	*D. neocordata*	***CG	Campo Grande/MGS/Brazil	Magalhães, E.	1973
*elliptica*	*D. emarginata*	***JD (14042-0841.09)	Vera Cruz/México	Markow, Th.	2005
*willistoni*	*D. willistoni*	***P9IDW	Pindorama/SP/Brazil	Penariol, L./Madi-Ravazzi, L.	2008

* = indicate Department of Zoology of University of São Paulo from Brazil;

** = indicate UC San Diego Drosophila Stock Center ordering number;

*** UNESP/IBILCE/São José do Rio Preto/SP/Brazil.

### Preparation of the terminalia for analysis

We used the Kaneshiro technique with some modifications [23] to prepare the terminalia for SEM analysis. The total number of terminalia and aedeagi prepared was approximately 600, and at least 20 terminalia of each strain was analyzed. The prepared parts of the terminalia and aedeagi were placed in modified Karnovsky fixative (2.5% glutaraldehyde, 2.5% formaldehyde in 0.05 M sodium cacodylate buffer, pH 7.2, with 0.001 M CaCl_2_) and were maintained at room temperature or in a refrigerator for at least 1 h. These aldehyde fixed samples were immersed in a solution of 1% osmium tetroxide in 0.05 M cacodylate buffer at pH 7.2 for 1 h at room temperature.

The samples fixed in OsO_4_ were subsequently washed with distilled water and then treated with increasing concentrations of acetone (30, 50, 70, 90 and 100%) for approximately 10 minutes in each solution, and three washes were performed in the 100% solution. At the end of the dehydration procedure, certain samples were passed through the critical point dried (K550, EMITECH) and mounted on a SEM stub with copper tape and sputter coated with gold/palladium. Other samples were directly mounted to the SEM stub and sputter coated without critical-point drying; these samples showed the same quality as those were critical-point dried. Images of the samples were imaged and analyzed using a scanning electron microscope (LEO 435 VPi SEM, Zeiss).

### Phylogenetic analysis

Nineteen diagnostic features of the terminalia and aedeagus were scored and used to form a data matrix for phylogenetic reconstruction of the *saltans* group (Table 2). All characters were treated as non-additive. A cladistic analysis using equal weights was undertaken with TNT software (Tree Analysis Using New Technology; [24]) including 1,000 iterations and using the following procedures: ratchet, drift, sectorial search, tree fusion and TBR max. Outgroup rooting [25] was implemented with *D. willistoni*. During the analysis, unambiguous optimization was chosen to describe the evolution of the assessed characters.

**Table 2:** Character matrix based on the terminalia and aedeagus of the *saltans* group species, employed in the cladistic analysis.

Characteristics/Species	*D.willistoni*	*D. prosaltans*	*D. lusaltans*	*D. saltans*	*D. austrosaltans*	*D. sturtevanti*	*D. dacunhai*	*D. milleri*	*D. parasaltans*	*D. neocordata*	*D. emarginata*
1. Fused Ventral Parameres	0	0	0	0	0	1	1	1	0	0	1
2. Middle Ventral Process	0	0	0	0	0	1	1	1	0	0	0
3. Scales in the Middle Ventral Process	0	0	0	0	0	0	1	1	0	0	0
4. Punctiform projection in the Aedeagus Apex	0	0	0	0	0	1	1	1	0	0	0
5. Ventral Parameres of Epandrium	0	0	0	0	0	1	1	1	2	0	0
6. Concaved Surtylus	0	1	1	1	1	0	0	0	0	1	1
7. Apical Crest of Aedeagus	0	1	1	1	1	0	0	0	0	0	0
8. Apical Crestl with Punctiform Projection	0	0	0	0	1	0	0	0	0	0	0
9. Groove in the Apical Crest	0	0	1	0	0	0	0	0	0	0	0
10. Bristles in the Aedeagus Apical Crest	0	0	0	1	0	0	0	0	0	0	0
11. Scales in the Aedeagus Apical Crest	0	1	1	0	1	0	0	0	0	0	0
12. Aedeagus Cape	0	1	1	1	1	0	0	0	1	0	0
13. Serrated Edge of Aedeagus Cape	0	1	1	2	1	0	0	0	1	0	0
14. Dorsal Cleft of Aedeagus	0	0	0	0	0	0	0	0	0	0	1
15. Frontal Processes of Aedeagus	0	1	0	1	1	1	1	1	1	0	1
16. Bipartite Aedeagus Apex	0	0	0	0	0	0	0	0	0	0	1
17. Surtylus Processes	0	0	0	0	0	0	0	0	0	1	0
18. Sickle-Shaped Processes of Aedeagus	0	0	0	0	0	0	0	0	0	1	0
19. Long Apodeme	0	1	1	1	1	0	0	0	1	0	0

Presence (1), presence with modifications (2) or absence (0).

## Results

**Figure 1:**
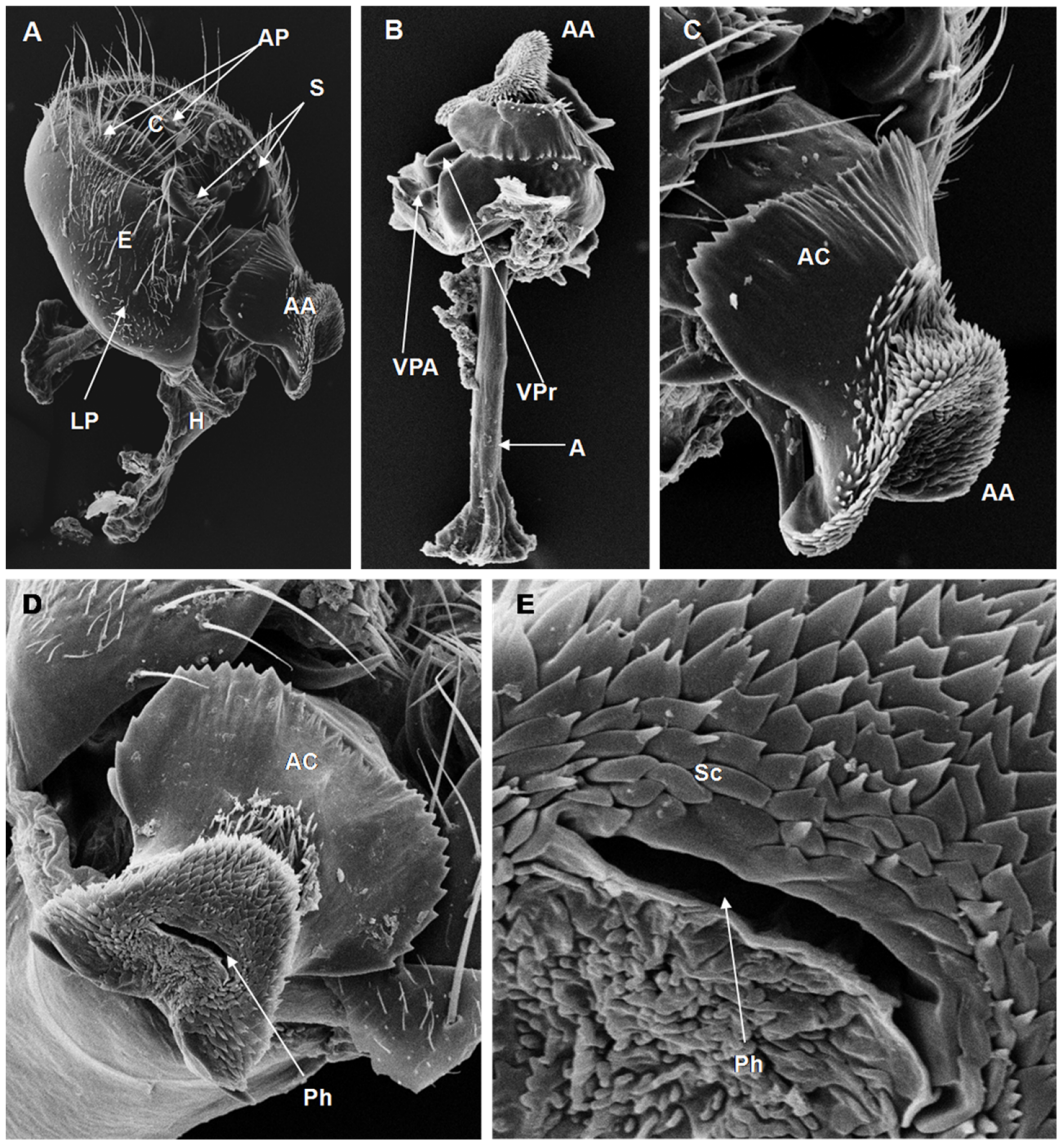
Scanning electron micrographs of the terminalia of *D. prosaltans* (PSR strain). (A) AA = aedeagus apex, AP = anal plate, C = cercus, E = epandrium, H = hypandrium, LP = lateral paramere, S = surstylus, (189× magnification); (B) AA = aedeagus apex, A = apodeme, AC = aedeagus cape, Sc = scales, VPA = ventral paramere of the aedeagus, VPr = ventral processes (395× magnification); (C) AA = aedeagus apex, AC = aedeagus cape, (253× magnification); (D) AC = aedeagus cape, Ph = phallotreme, (376× magnification); (E) Ph = phallotreme, Sc = scales, (1610× magnification).

**Figure 2:**
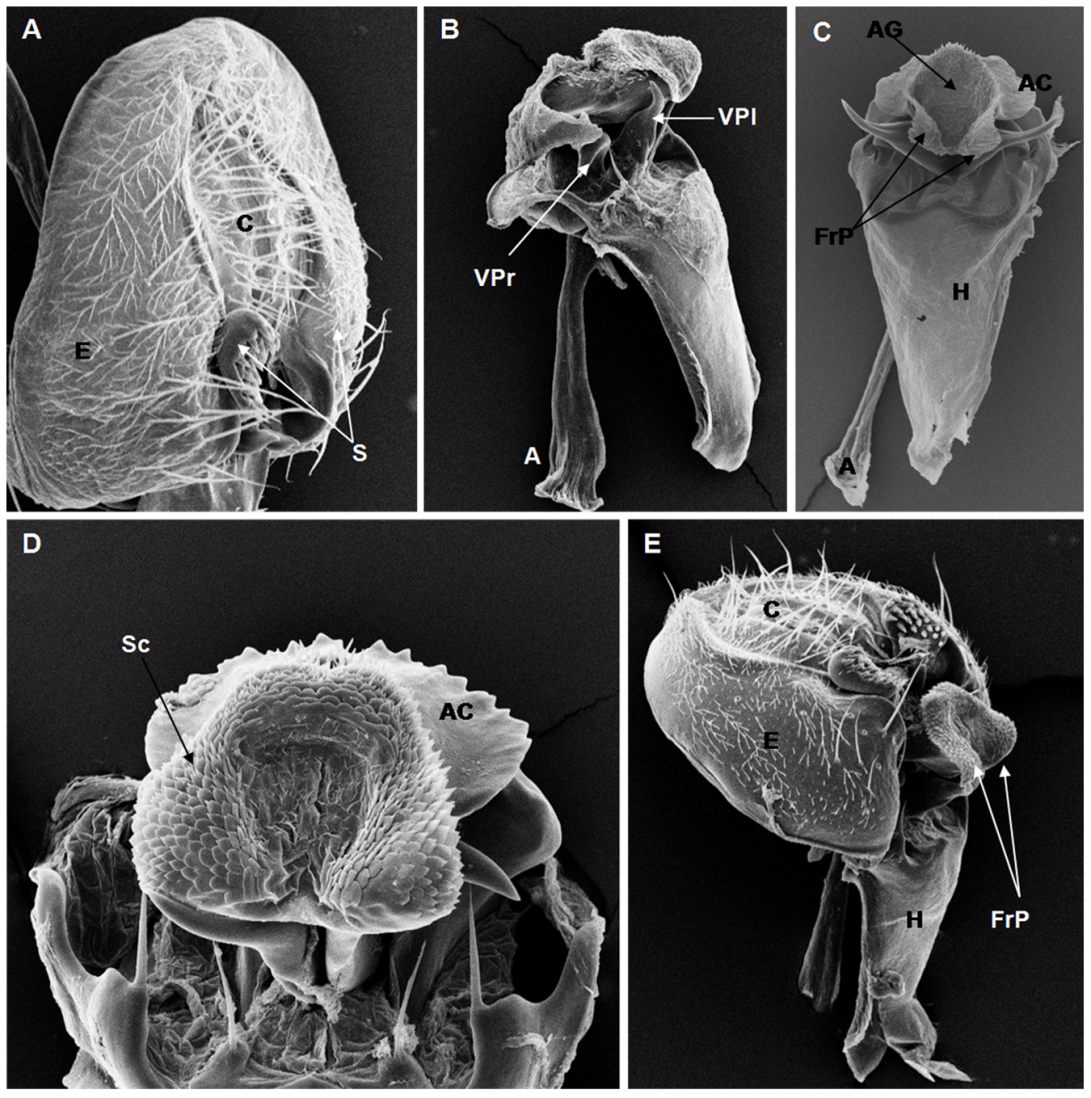
Scanning electron micrographs of *D. lusaltans* terminalia. (A) C = cercus, E = epandrium, S = surstylus, (186× magnification); (B) VPl = ventral prolongation, VPr = ventral processes, A = apodeme (198× magnification); (C) AC = aedeagus cape, AG = apical grove, FrP = frontal processes, H = hypandrium, (180× magnification); (D) AC = aedeagus cape, Sc = scales, (466× magnification); (E) C = cercus, E = epandrium, H = hypandrium, FrP = frontal processes, (150× magnification).

**Figure 3:**
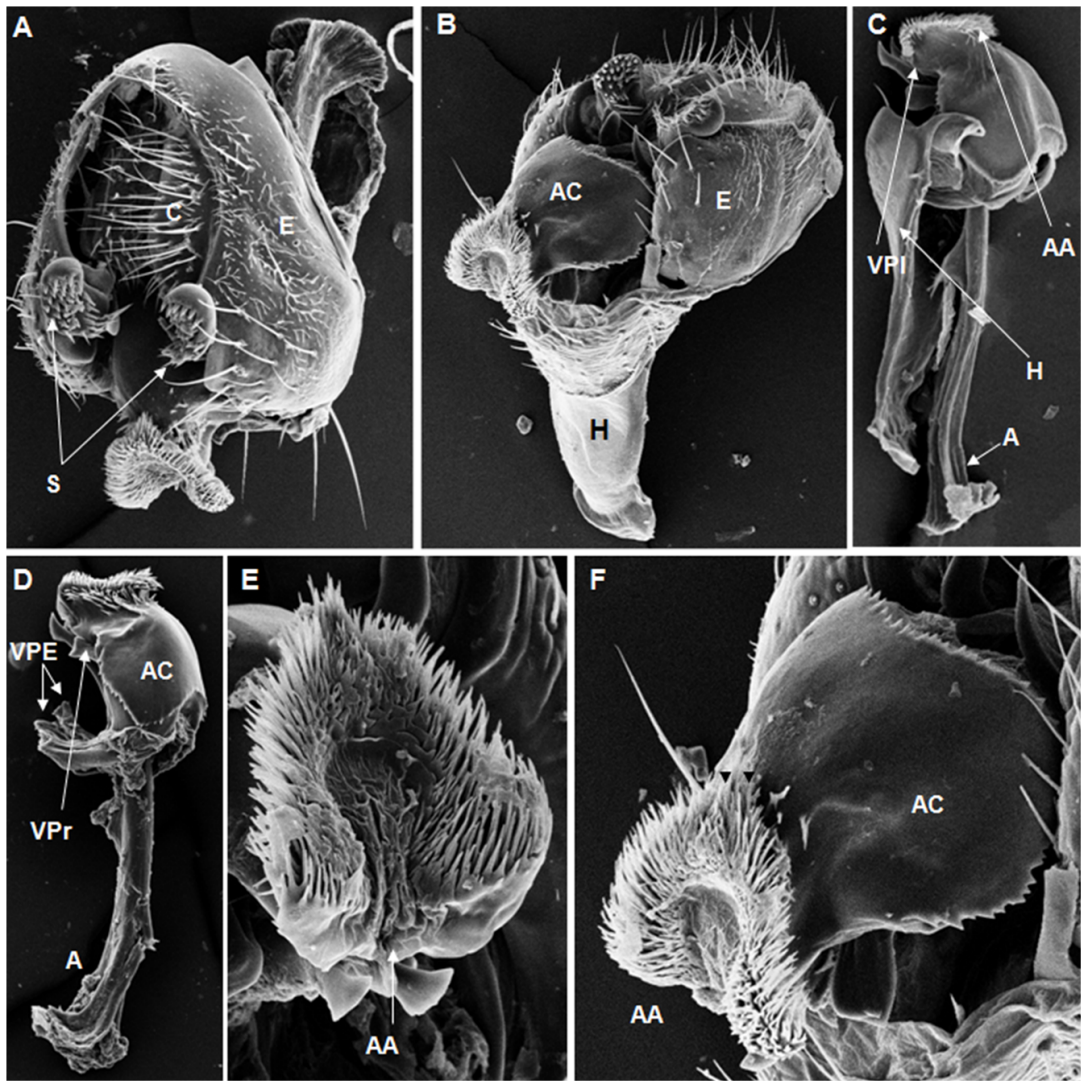
Scanning electron micrographs of *D. saltans* terminalia. (A) C = cercus, E = epandrium, S = surstylus, (222× magnification); (B) AC = aedeagus cape, E = epandrium, H = hypandrium, (170× magnification); (C) AA = aedeagus apex, VPl = ventral prolongation, VPr = ventral processes, (200× magnification); (D) A = apodeme, AC = aedeagus cape, VPA = ventral paramere of aedeagus, (241× magnification); (E) AA = aedeagus apex, (530× magnification); (F) AA = aedeagus apex, AC = aedeagus cape, setae on the aedeagus apex (arrow heads), (274× magnification).

**Figure 4:**
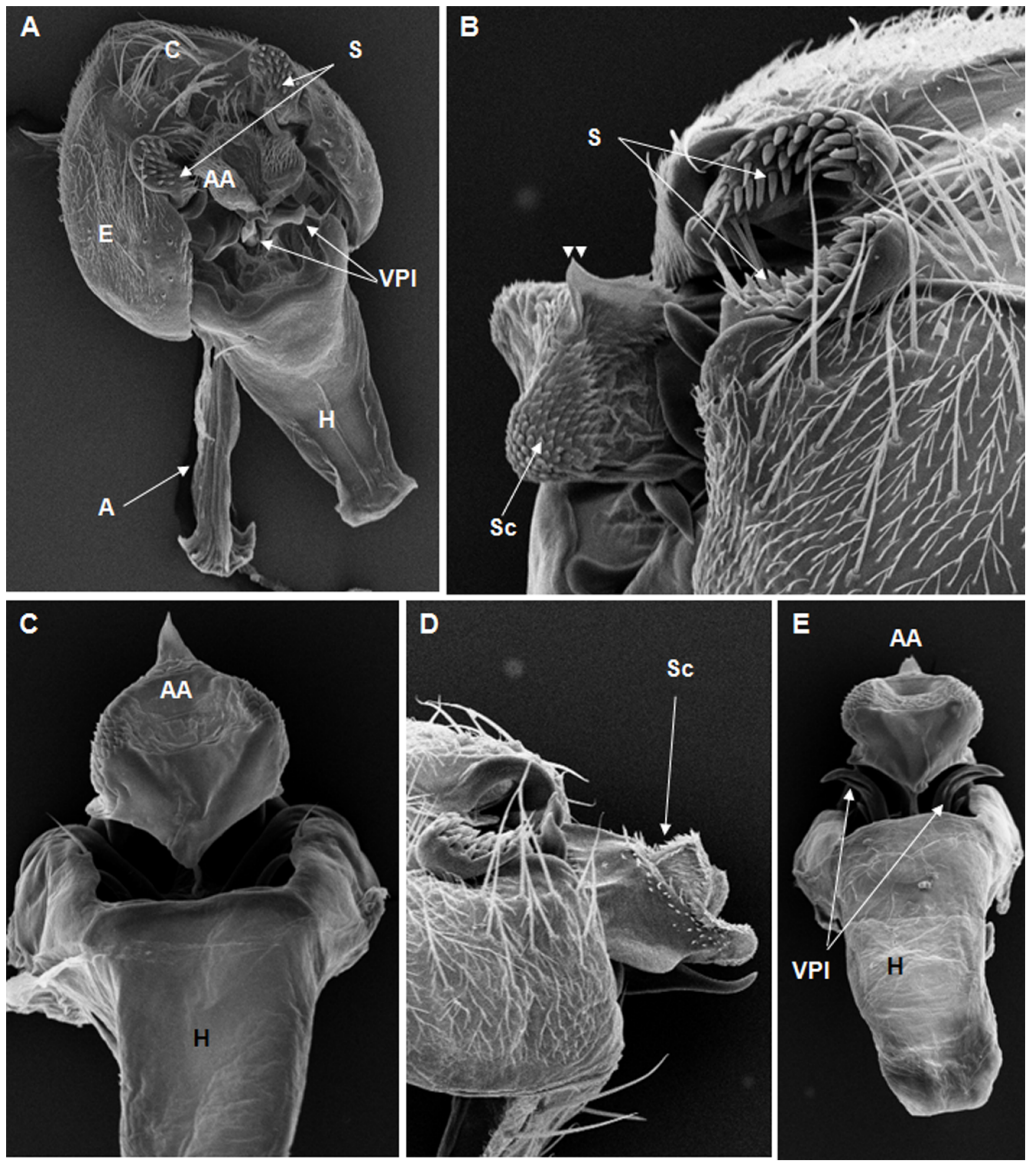
Scanning electron micrographs of *D. austrosaltans* terminalia. (A) A = aedeagus, AA = aedeagus apex, C = cercus, H = hypandrium, E = epandrium, VPl = ventral prolongations, (179× magnification); (B) S = surstylus, Sc = scales, punctiform projection on the aedeagus apex (arrow heads), (351× magnification); (C) AA = aedeagus apex, H = hypandrium, (432× magnification); (D) Sc = scales, AA = aedeagus apex, AC = aedeagus cape, (466× magnification); (E) A = apodeme, AC = aedeagus cape, (322× magnification).

**Figure 5:**
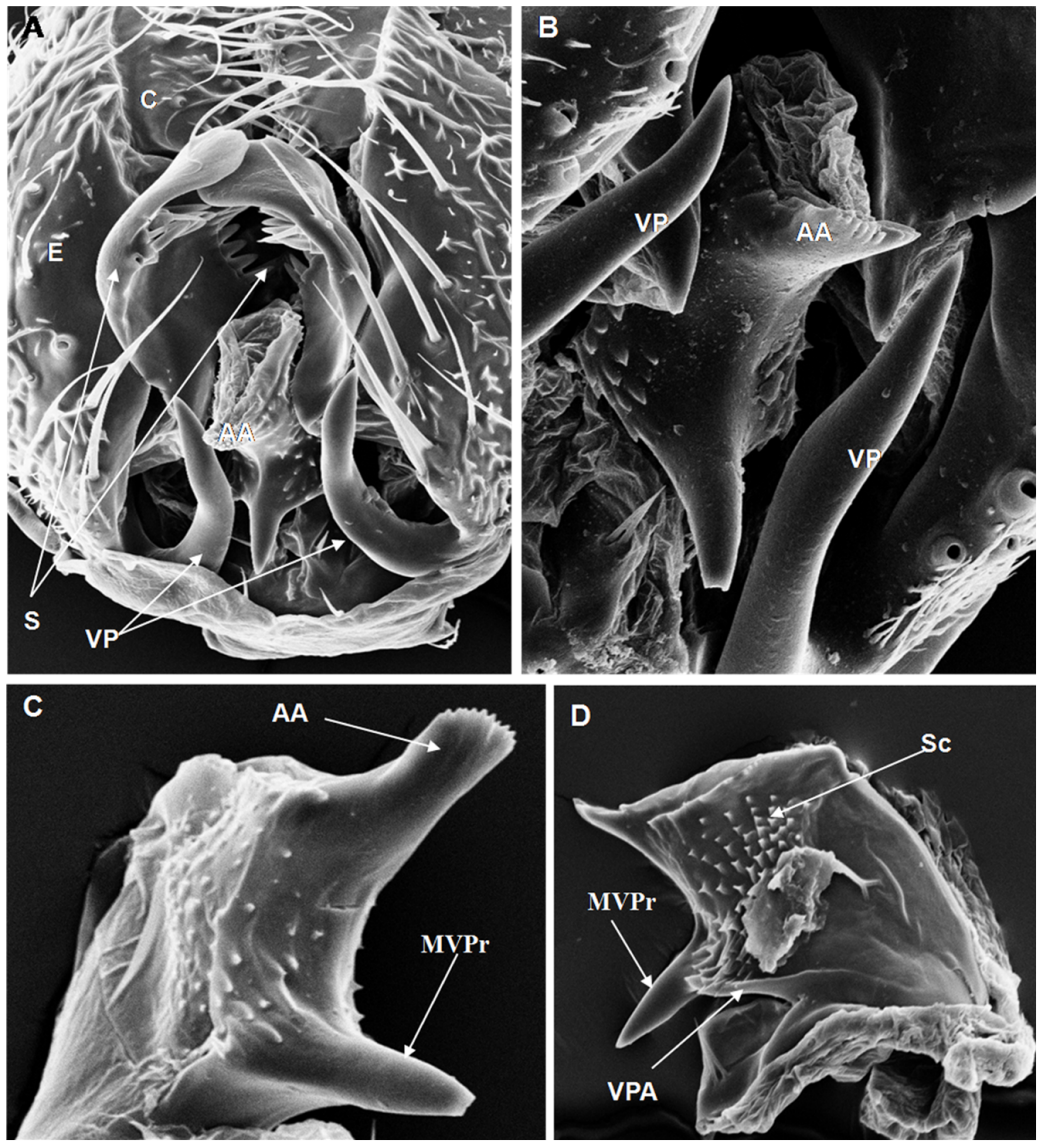
Scanning electron micrographs of *D sturtevanti* terminalia (TUR strain). (A) C = cercus, E = epandrium, VP = ventral paramere, S = surstylus (400× magnification); (B) AA = aedeagus apex, VP = ventral paramere (747× magnification); (C) AA = aedeagus apex, MVPr = middle ventral process, (817× magnification); (D) MVPr = middle ventral process, VPA = ventral paramere of the aedeagus, Sc = scales, (578× magnification).

**Figure 6:**
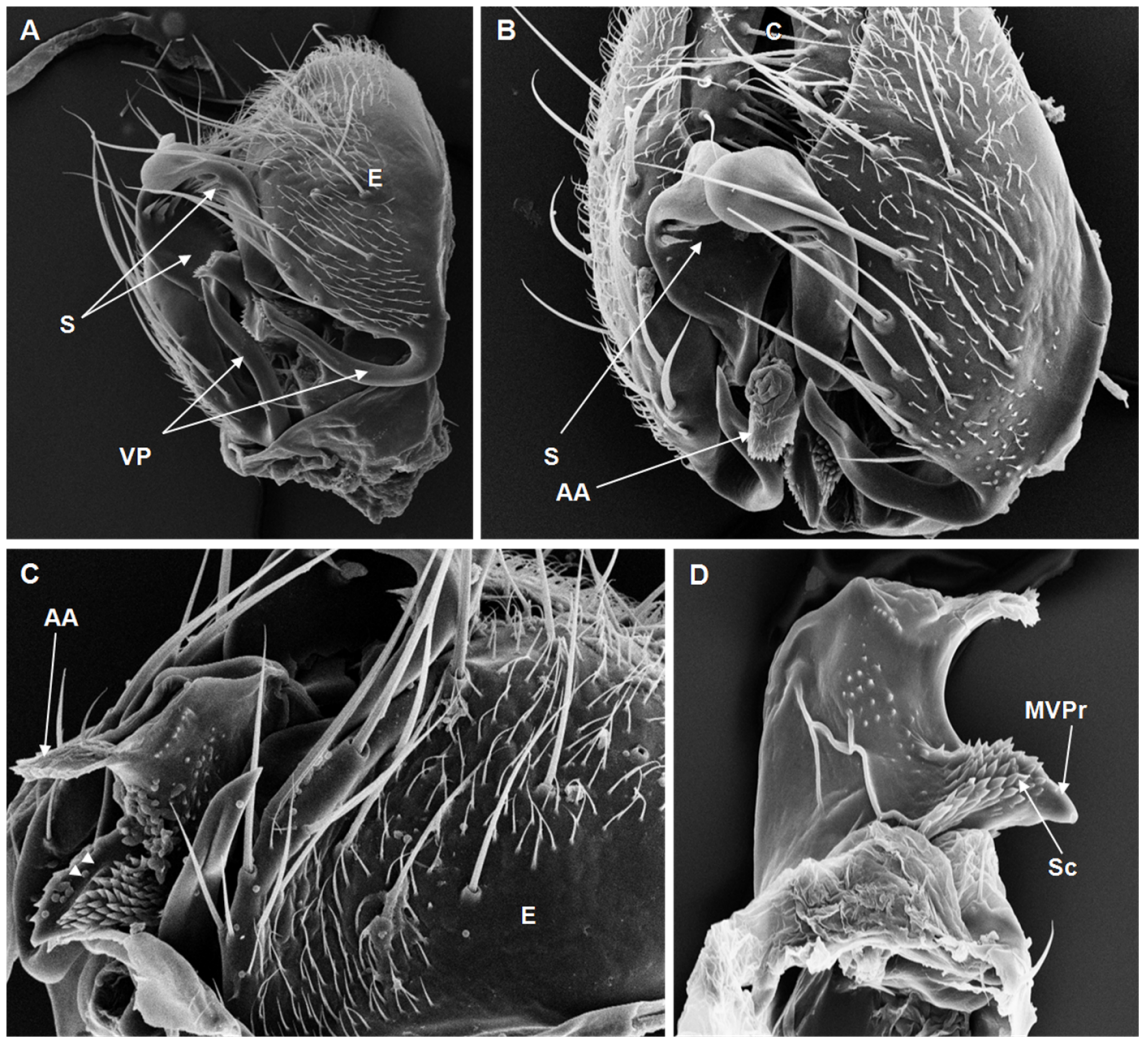
Scanning electron micrographs of *D. dacunhai* terminalia. (A) S = surstylus, VP = ventral paramere, E = epandrium, (256× magnification); (B) AA = aedeagus apex, C = cercus, E = epandrium, S = surstylus, (350× magnification); (C) AA = aedeagus apex, E = epandrium, groove on the MVPr (arrow heads), (420× magnification); (D) MVPr = middle ventral process, Sc = scales, (582× magnification).

**Figure 7:**
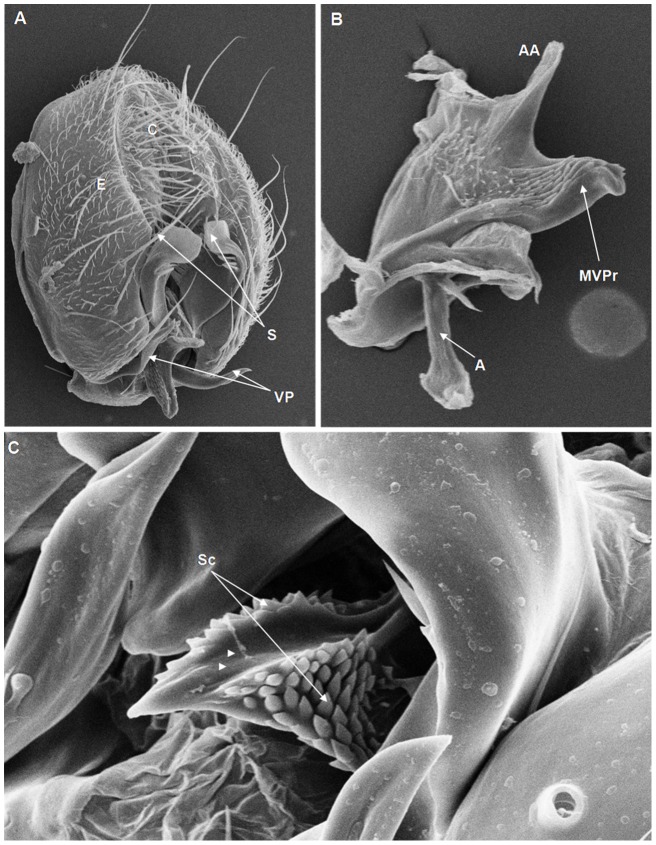
Scanning electron micrographs of *D. milleri* terminalia. (A) C = cercus, E = epandrium, S = surstylus, VP = ventral paramere, (239× magnification); (B) AA = aedeagus apex, MVPr = middle ventral process, A = apodeme, (582× magnification); (C) C = cercus, Sc = scales, groove on the MVPr (arrow heads), (1160× magnification).

**Figure 8:**
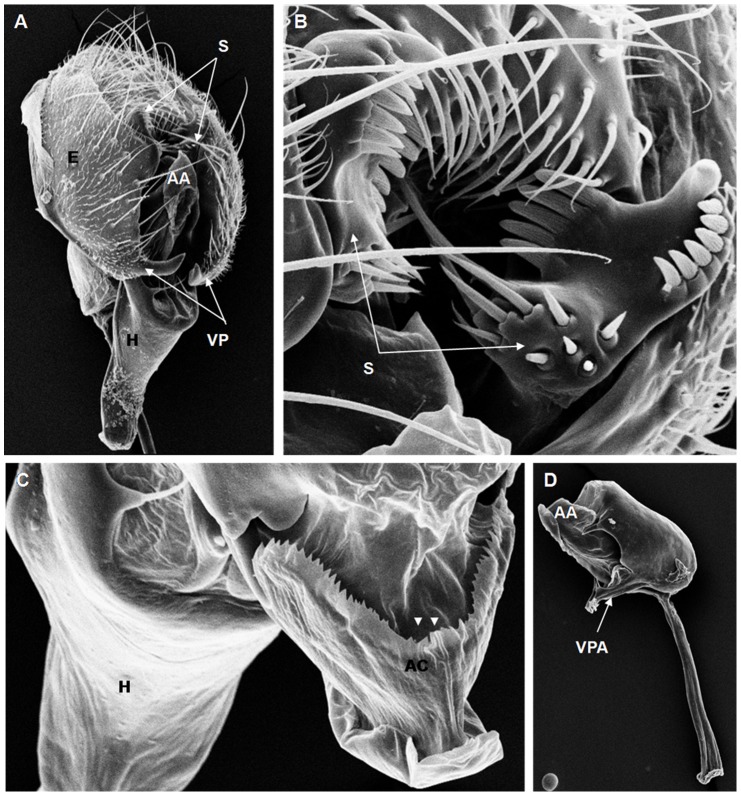
Scanning electron micrographs of *D. parasaltans* terminalia. (A) AA = aedeagus apex, C = cercus, E = epandrium, H = hypandrium, S = surstylus, VP = ventral paramere, (152× magnification); (B) S = surstylus, (858× magnification); (C) H = hypandrium, AC = aedeagus cape, recesses in aedeagus (arrow heads), (532× magnification); (D) A = apodeme, AA = aedeagus apex, VPA = ventral paramere of the aedeagus, (206× magnification).

**Figure 9:**
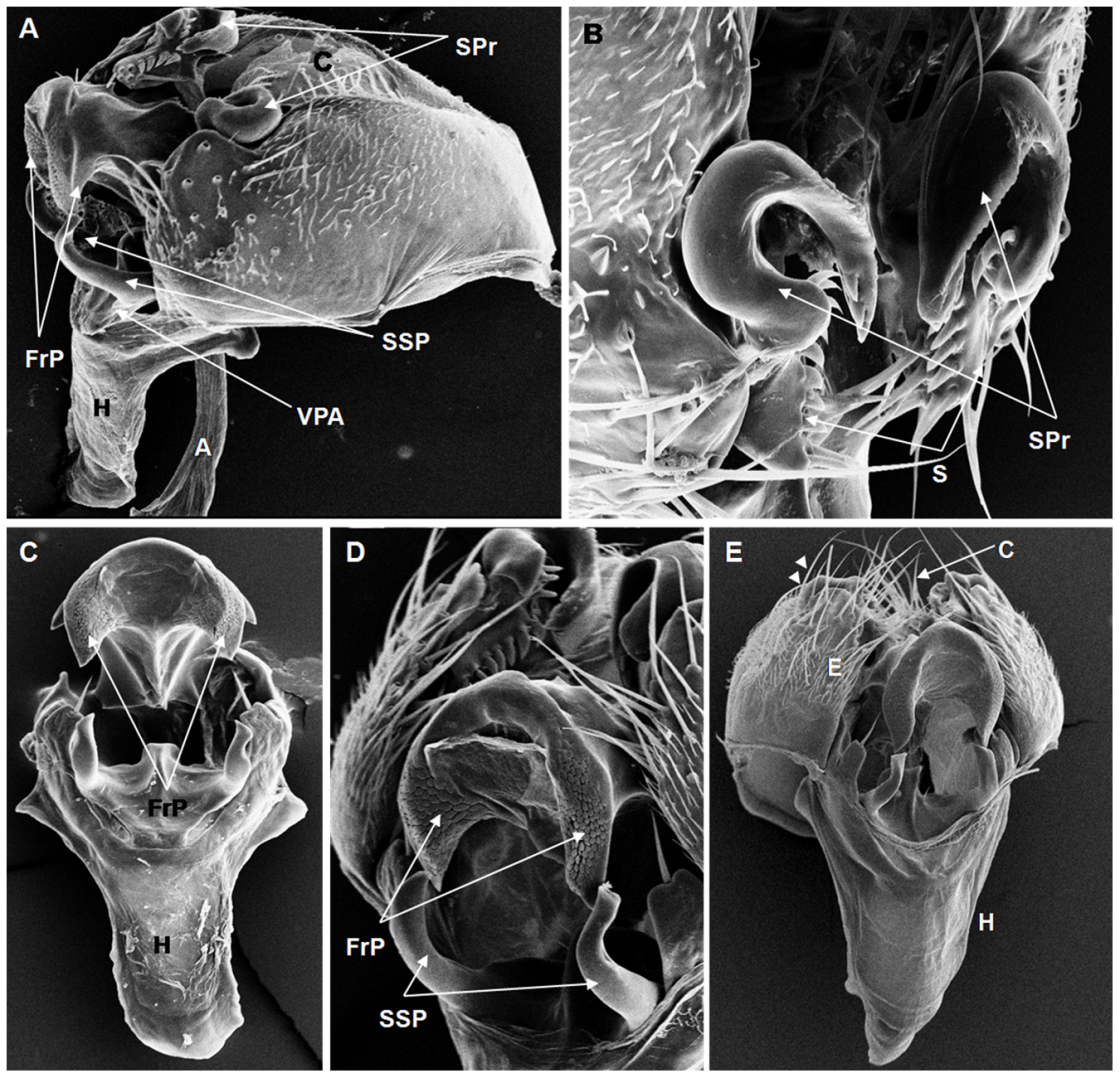
Scanning electron micrographs of *D. neocordata* terminalia. (A) A = apodeme, C = cercus, FrP = frontal process, SPr = surstylus processes, SSP = sickle-shaped processes, H = hypandrium, VPA = ventral paramere of the aedeagus, (161× magnification), (B) S = surstylus, SPr = surstylus processes, (578× magnification); (C) FrP = frontal process, H = hypandrium, (248× magnification); (D) FrP = frontal process, SSP = sickle-shaped processes, (318× magnification); (E) C = cercus, E = epandrium, H = hypandrium, epandrium bristles (arrow heads), (202× magnification).

**Figure 10:**
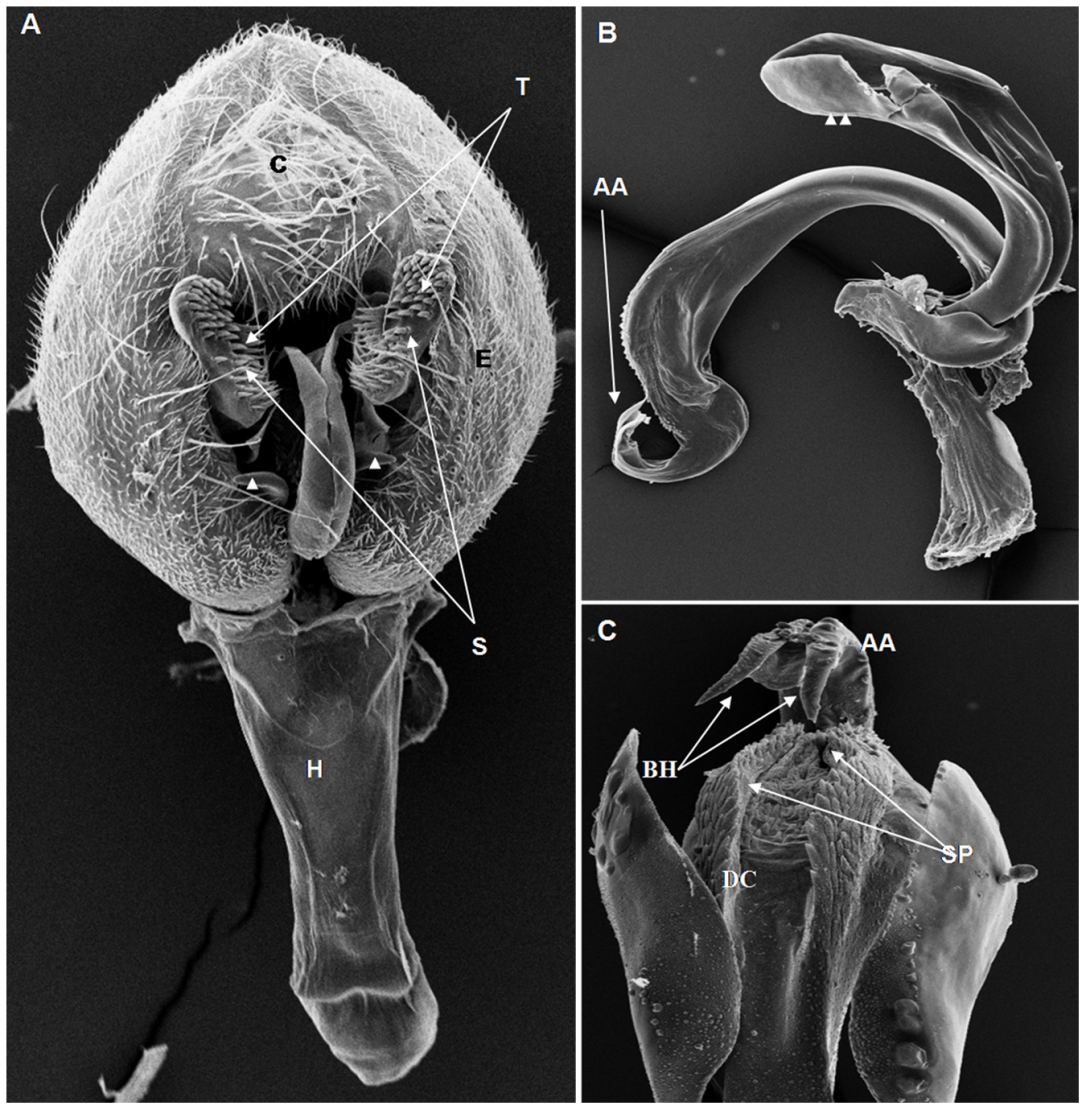
Scanning electron micrographs of *D. emarginata* terminalia. (A). C = cercus, E = epandrium, S = surstylus, T = primary and secondary teeth, H = hypandrium, cuticular hook of the epandrium (arrow heads), (141× magnification); (B) AA = aedeagus apex, VPA = ventral paramere of the aedeagus, lateral processes of the aedeagus (arrow heads), (130× magnification); (C) AA = aedeagus apex, BH = bipartite hook, DC = dorsal cleft, SP = serrated plates, (309× magnification).

**Figure 11:**
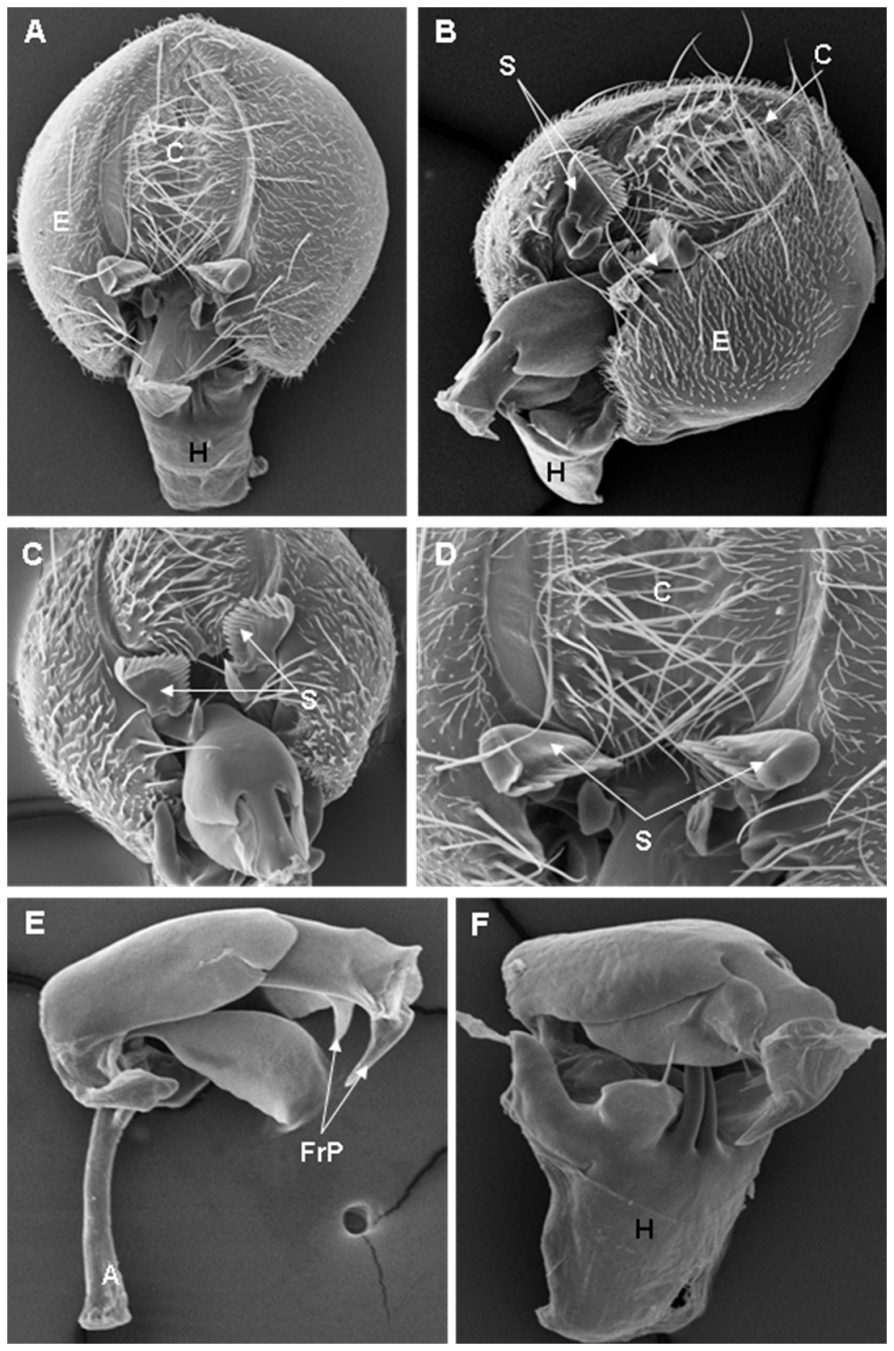
Scanning electron micrographs of *D. willistoni* terminalia. (A) C = cercus, E = epandrium, H = hypandrium, (171× magnification); (B) C = cercus, E = epandrium, H = hypandrium, S = surstylus,(178× magnification); (C) S = surstylus, (337× magnification); (D) C = cercus, S = surstylus, (510× magnification); (E) FrP = frontal, (268× magnification); (F) H = hypandrium, (304× magnification).

The 19 characteristics of the male terminalia of species from the *saltans* group that were analyzed by SEM are described in Table 2 and illustrated in Figures 1 to 11. The terminology applied for features that had previously been described based on camera lucida drawings was maintained as reported in previous works [1], [2], [26], [27], [28], [29] with certain modifications. The description of the main characteristics of the terminalia and aedeagus analyzed in this study is presented in Table S1 (Supporting Information).

On the basis of the present analysis, we characterized the subgroups and species as follows: *saltans* subgroup: The epandrium (E) is an antero-inferior region that is highly angular, preceding the two recesses similar to curved gloved fingers (Figures 1 A; 2 A; 3 A; 4 A). The hypandrium (H) is elongated with two pairs of extensions, one of which is internal and the other external (Figures 1A; 2B, C, E; 3B; 4A, C). *D. prosaltans* (Figure 1): The surstyli (S) are located above the recesses with the form of gloved fingers and just below each side of the epandrium, with a long decasternum (D) between them and with primary and secondary teeth present; the anal plates (AP) exhibit a U-shaped contour (Figure 1A). The aedeagus (A) is characterized by ventral processes (VPr) (Figure 1B) similar to curved gloved fingers, with an apical region (AA) (Figure 1A) that is strongly sclerotized and contains a serrated crest covered with scale-like structures (Sc) (Figure 1 B–C) that extends to the mid-dorsal region of the aedeagus in the form of a cape (AC) (Figure 1B–D), which is serrated and sclerotized at its edge and displays ventral parameres (VPA) (Figure 1C–D) and ventral prolongations (VPl) (Figure 1B). Additionally, it presents an apodeme (A) without pronounced curvature and triangular ends (Figure 1B). The phallotreme (Ph) is a fissure-like structure located at the apex of the aedeagus that can be observed only in SEM analysis (Figure 1D–E). *D. lusaltans* (Figure 2): The surstyli exhibit morphology similar to a bean seed (Figure 2A, E). The aedeagus is characterized by the presence of an apical crest that is very similar to that of *D. prosaltans*; however, *D. lusaltans* displays an apical groove (AG) that isolates two lateral protuberances that end in two frontal processes (Figure 2C). The aedeagus apex has scales and an aedeagus body with a serrated edge (Figure 2D). The ventral processes are longer and thinner and cover the entire length of the ventral region of the aedeagus (Figure 2C). The apodeme is straight and long (Figure 2B–C). *D. saltans* (Figure 3): The surstyli exhibit morphology similar to a bean seed (Figure 3A–B); (Figure 3B). The aedeagus is characterized by a flattened apical region, with long ventral extensions derived from the insertion of the apodeme that extend through the length of the ventral region of the aedeagus, curving at its end. The ventral parameres of the aedeagus extend from the insertion of the apodeme to the dorsal medial region of the aedeagus (Figure 3C–E). The cape of the aedeagus has a strongly serrated edge that can be observed in detail via SEM (Figure 3B, D, F). In contrast to the other *saltans* subgroup species, which have scales on the aedeagus apex, this species displays setae in this region (Figure 3B, E, F). The apex of the aedeagus exhibits a punctiform projection in the posterior portion consisting of numerous long setae (Figure 3E, arrowheads). *D. austrosaltans* (Figure 4): The surstyli show morphology similar to a bean seed (Figure 4A). The hypandrium differs from that of other species of the *saltans* group due to the presence of square edges rather than rounded edges and presents a ventral prolongation (VPl) extending the entire length of the aedeagus ventral region, which is parallel in its initial portion and divergent in the terminal portion (Figure 4A). The aedeagus is characterized by the presence of a drop-shaped crest at the apex with a punctiform dorsal protuberance that is more pronounced than in the previously described species (Figure 4B, arrowhead; Figure 4C). The apex of the aedeagus is covered with scales, as observed in *D. prosaltans* and *D. lusaltans* (Figure 4B, D). *sturtevanti* subgroup: The epandrium displays ventral parameres and anal plates forming a U-shaped contour (Figure 5A, 6B, 7A). *D. sturtevanti* (Figure 5): The surstyli are large and concave and are joined by a small decasternum; primary and secondary teeth (T) are present (Figure 5A). The aedeagus is characterized by the presence of fused ventral parameres, where the middle ventral process (MVPr) exhibits a pointed apical region and has the appearance of a duck's beak. The cape of the aedeagus is absent, and the apodeme is short and sinuous (Figure 5C–D). Structures consisting of similar scales (Sc) near the apex of the aedeagus could be visualized only in the SEM analysis and there were fewer of them than observed in *D. dacunhai* and *D. milleri*. No difference in the morphology of the terminalia or aedeagus was observed among the analyzed strains of *D. sturtevanti*. *D. dacunhai* (Figure 6): The surstyli are large and concave and are joined by a small decasternum (Figure 6A–B). The aedeagus is characterized by the presence of fused ventral parameres with the appearance of a duck's beak (Figure 6C–D); scales are present in the middle ventral process (Figure 6C–D), which is wider and more curved than that of *D. sturtevanti* and presents pointed scales and a groove that has not been described previously (Figure 6C, arrowhead). *D. milleri* (Figure 7): The surstyli are large and concave (Figure 7A). The aedeagus is characterized by fused ventral parameres that combine with the middle ventral process, resulting in a duck-beak-like appearance (Figure 7B), and presents a groove in the upper portion of the middle ventral process (Figure 7C, arrow head), which is covered by scales that are close to the surface of the process (Figure 7B–C). *parasaltans* subgroup: *D. parasaltans* (Figure 8): The epandrium displays ventral parameres similar to those of the *sturtevanti* subgroup and exhibits a cercus with many bristles, mainly in the region just above the surstyli (Figure 8A). The surstyli are concave, with a very different morphology; they are characterized by the presence of four to five teeth on the inside edge and six to seven teeth on the outer edge (Figure 8B). The aedeagus has a dorsal part formed by a single structure without recesses and displays extensions projecting laterally that contain spiniform structures. The apodeme is long and straight, not sinuous (Figure 8B), and the ventral parameres are short and thin with two spiniform projections on the apical regions. The cape of the aedeagus presents a serrated edge along its entire dorsal length (Figure 8C). *cordata* subgroup: *D. neocordata* (Figure 9): The epandrium does not have ventral processes but exhibits long and short bristles in the basal region. The antero-inferior region of the epandrium is situated at nearly a right angle and presents anal plates forming a U-shaped contour (Figure 9A–B). The surstyliare formed by cuticular plates that are inserted into five to six primary teeth and six long bristles; large processes are present (SPr) along with protruding structures with the appearance of gloved fingers, which are diagnostic characteristics of the species (Figure 9 A–B). The apodeme of the aedeagus is short and thick (Figure 9A). The frontal processes (FrP) at the bottom of the apex are similar to claws (Figure 9C–E). The ventral parameres of the aedeagus are long and thin and bifurcate in the middle region, giving rise to cuticular prolongations that extend to the ventral region of the body of the aedeagus and end in the sickle-shaped processes (SSP). The surstylus processes and the sickle-shaped processes are characteristic of this species (Figure 9A–D). *elliptica* subgroup: *D. emarginata* (Figure 10): The hypandrium is elongated and thin (Figure 10 A). The epandrium has two pairs of cuticular hooks on the inside edge of its angular lower region (Figure 10A, arrow heads). The aedeagus is extremely large compared with the other analyzed species of the *saltans* group and appears sickle-like. The central axis of the aedeagus has a slit in its dorsal region (FD) and ends in a hook (BH) that branches into two pointed ends (Figs. 10B–C). Its apodeme is wide and short (Figure 10B), and its lateral processes are large and long and follow almost the entire length of the central axis (Figure 10B, arrow heads). The fused ventral paramere is similar to that of the *sturtevanti* subgroup species, and the dorsal slit is covered by plates with serrated edges, which is another feature that was detailed only in the SEM analysis (Figure 10B–C). *D. willistoni* (outgroup) (Figure 11): The hypandrium is short and wide with a quadrangular morphology (Figure 11A, B). The surstyli are concave and are formed by two parts, with a larger apical part that contains primary teeth and a smaller part that is fused to the larger part and contains secondary teeth (Figure 11B–D). The dorsal portion of the aedeagus is formed by a plate that extends on both its sides (Figure 11C, arrowhead). In the dorsal portion of this plate, there is a tubular projection that extends along the aedeagus body and ends in two frontal projections (Figure 11 C, F). The ventral parameres of the aedeagus are short and thick, and the apodeme is short and thin (Figure 11 E).

Among the described features, nine could be observed only through SEM analysis: 1. the scales on the middle ventral process in *D. dacunhai* and *D. milleri* (Figure 6C–D); 2. the punctiform projection on the aedeagus apex in the three species of the *sturtevanti* subgroup (Figure 5C–D, Figure 6C–D); 3. the apical crest with the punctiform projection in *D. austrosaltans* (Figure 4B); 4. the groove on the apical crest in *D. lusaltans* (Figure 2C–D); 5. the bristles on the apical crest of the aedeagus in *D. saltans* (Figure 3E–F); 6. the scales on the apical crest of the aedeagus in *D. prosaltans*, *D. lusaltans* and *D. austrosaltans* (Figure 1C–E; Figure 2D; Figure 4B, D); 7. the serrated edge of the aedeagus cape in all species of the *saltans* subgroup and *D. parasaltans* (*parasaltans* subgroup) (Figure 1C–D; Figure 2D; Figure 3B, C, D, F; Figure 4D–E; Figure 8C); 8. the dorsal cleft of the aedeagus; and 9. the bipartite aedeagus apex in *D. emarginata* (Figure 10C) (table 2).

Some characteristics were shared by different subgroups, whereas others were subgroup-specific or species-specific. Five features were specific to the *saltans* subgroup (the apical crest of the aedeagus, the apical crest with a punctiform projection, the groove on the apical crest, the bristles on the aedeagus apical crest and the scales on the aedeagus apical crest); two were specific to the *sturtevanti* subgroup (the middle ventral process, the scales on the middle ventral process and the punctiform projection on the aedeagus apex magnification); two were specific to the species *D. neocordata*, representing the *cordata* subgroup (the surstylus processes and sickle-shaped processes of the aedeagus); and two were specific to the species *D. emarginata*, representing the *elliptica* subgroup (the dorsal cleft of the aedeagus and the bipartite aedeagus apex). *D. parasaltans*, representing the *parasaltans* subgroup, did not show subgroup-specific or species-specific features (Table 2).

Species-specific features were observed in *D. lusaltans* (the groove on the apical crest), *D. saltans* (the bristles on the aedeagus apical crest), *D. neocordata* (the surstylus processes and sickle-shaped processes of the aedeagus) and *D. emarginata* (the dorsal cleft of the aedeagus and the bipartite aedeagus apex) (Table 2).

Subgroup-specific features that are shared among species were found in the *saltans* subgroup (the aedeagus cape, serrated edge of the aedeagus cape, frontal processes of the aedeagus and long apodeme) and the *sturtevanti* subgroup (the fused ventral parameres, ventral parameres of the epandrium and concave surstyli), which were the only subgroups for which more than one species was available for analysis (Table 2).

Features shared between subgroups were observed in the *D. sturtevanti* subgroup and *D. parasaltans* (the ventral parameres of the epandrium and concave surstyli); the *D. sturtevanti* subgroup and *D. emarginata* (fused ventral parameres); the *saltans* subgroup and *D. parasaltans* (the aedeagus cape, serrated edge of the aedeagus cape and long apodeme); and the *D. saltans* subgroup and *D. neocordata* (the frontal processes of the aedeagus; Table 2).

**Figure 12:**
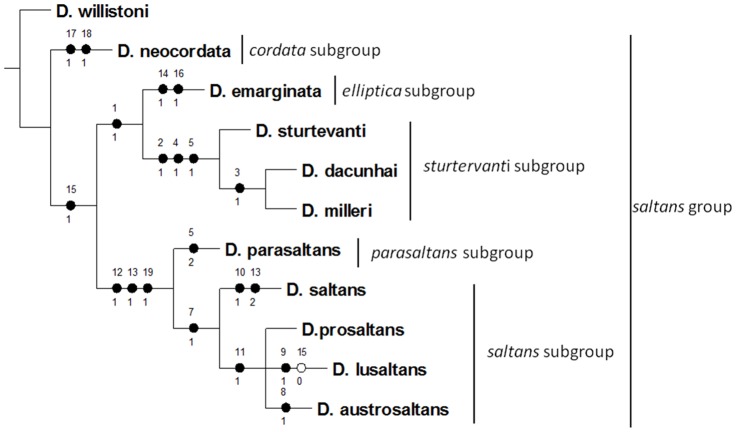
Phylogenetic tree of the subgroups of *saltans* group. The single most parsimonious cladogram for the *saltans* group based on 19 morphological characters, as described in this work. Length = 25, CI = 87 and RI = 90. Black circles represent synapomorphies, white circles represent homoplasies. Numbers above and below the circles represent character number and character state, respectively, as depicted in Table 2.

The nineteen characters evaluated in detail via SEM were used to construct a matrix for phylogenetic analysis of the species of the *saltans* group, which is shown in Table 2. Cladistic analysis using only the data obtained in this study recovered a single most parsimonious tree with a length of 25 steps (CI = 87; RI = 90) and recovered the monophyly of the *saltans* group. The consensus tree indicated that the *cordata* subgroup is the most basal lineage and the *saltans* subgroup is the most apical lineage (Figure 12).

## Discussion

The application of SEM to the study of male terminalia in the *Drosophila saltans* group provided tools for improving the recognition of species and for developing a phylogenetic tree based on new morphological markers. Nine of the 19 structures selected for analysis were described for the first time in the present study, whereas the remaining structures were assessed by detailing known structures that were previously analyzed via light microscope.

Some of our findings distinguished sibling species in the *saltans* and in the *sturtevanti* subgroups more precisely. In the *saltans* subgroup, a species-specific structure allowed differentiation of *D. saltans* from *D. prosaltans, D. austrosaltans* and *D. lusaltans*. *Drosophila lusaltans* and *D. austrosaltan* also had species-specific characters. In the *sturtevanti* subgroup, by the same criterion, *D. sturtevanti* was differentiated from *D. dacunhai* and *D. mulleri*. *D. parasaltans*, *D. neocordata* and *D. emarginata*, the unique available species representing the subgroups *parasaltans*, *cordata* and *elliptica*, respectively, presented characters that allowed differentiation among them and from the species in the subgroups *saltans* and *sturtevanti*.

It is interesting to compare the morphological data on the aedeagus with data on the reproductive isolation of the same species [29], [30], [31]. In the present phylogenetic analysis, *D. saltans* differed from *D. lusaltans*, *D. austrosaltans* and *D. prosaltans*. However, data on reproductive isolation [29] indicated that *D. austrosaltans* is completely reproductively isolated from these species at the premating level. In contrast, *D. prosaltans*, *D. saltans* and *D. lusaltans* are not completely reproductively isolated from each other, as *D. lusaltans* can produce fertile F1 offspring with *D. prosaltans* and with *D. saltans*. Additionally, *D. prosaltans* can be intercrossed with *D. saltans* yielding fertile F1 females and males in one direction and fertile females and sterile males in the other direction. The *sturtevanti* subgroup, whose species show great similarity with regard to the male terminalia produced a low number of hybrid progeny in the intercrosses of *D. milleri* with *D. sturtevanti* or *D. magalhaesi*, generating a phylogenetic sequence in which *D. magalhaesis* was the most basal species, followed, in sequence, by *D. milleri*, *D. sturtevanti* and *D. dacunhai* [31]. In the tree proposed in the present paper, *D. milleri* and *D. dacunhai* were considered sister taxa. These incongruent results are compatible with the finding that premating mechanisms (probably sexual isolation) are predominantly involved in the reproductive isolation of species in the *saltans* group [29], [31].

In general, phylogenetic relationships within the *saltans* group based on different markers have been found to be incongruent, but monophyly of the group has always been recovered [4], [7], [17]. The same occurred in the present study. However, comparison of the evolutionary relationships based on SEM analysis with previous data revealed high consistency with the sequential order of the subgroups obtained using morphological and biochemical data [2],[3]. According to the results of these studies, the *cordata* and *elliptica* subgroups are the most basal, followed by *sturtevanti* and *parasaltans*, with the *saltan*s subgroup being the most derived.

Unlike, as observed regarding the reproductive isolation already mentioned, comparison of the present phylogenetic data with other studies using different markers indicated only partial concordance. For example, one molecular phylogenetic study using the genes that code for ADH, ITSI, COI and COII [10] produced phylogenetic relationships that were predominantly incongruent relative to those of Magalhães [1], Throckmorton and Magalhães [2] and the present SEM data. O'Grady et al. [10] suggested that unresolved branching patterns were due to the relatively recent divergence of these species and conflicting information from each locus. In both O'Grady et al. [10] and the present study, *Drosophila prosaltans* and *D. austrosaltans* were considered to be closer to each other than to *D. saltans*.

When our cladogram was compared with that of Yassin [4], which was based on morphological markers, the main difference observed was related to the basal group, which was the *cordata* clade in the present work and the *sturtevanti* subgroup in Yassin's study. In both studies, the *parasaltans* and *saltans* subgroups appeared as sister clades. Yassin [4] suggested that the discrepancies in molecular phylogenies in the *saltans* group may have resulted from a characteristic shift of codon bias in Sophophorans from the New World. Yassin [32] found that different morphological characteristics provide varying signals at different phylogenetic scales. Therefore, molecular and morphological data must be employed to better understand the taxonomy and phylogeny of these organisms.

The analysis of datasets based on phylogenetic relationships determined using different markers for species in the *saltans* group suggests that increased taxon sampling and identification of more informative markers are necessary to better resolve the phylogeny of this group. The present results indicate that SEM characteristics may be useful in the species recognizing and useful as a marker for attempting to clarify the evolutionary history of the group.

## Supporting Information

Table S1 **Description of the structures of the terminalia and the aedeagus analysed by SEM (Scanning Electron Microscopy).**
(DOC)
