# Supplementary material for: Scanning Electron Microscopy of Male Terminalia and Its Application to Species Recognition and Phylogenetic Reconstruction in the Drosophila saltans Group
Source: PLoS One. 2014 Jun 10;9(6):e97156. doi: 10.1371/journal.pone.0097156 (PMC4051629; doi:10.1371/journal.pone.0097156)
Supplement: Table S1 — Description of the structures of the terminalia and the aedeagus analysed by SEM (Scanning Electron Microscopy). (DOC) [file pone.0097156.s001.doc]

| Structures | Description |
| --- | --- |
| Apodeme (A) | Appendix that is connected or fused at the base of the aedeagus. |
| Aedeagus Apex (AA) | Apical region of the aedeagus, characterised by the presence of scales and by the insertion of the aedeagus cover. |
| Aedeagus Cape (AC) | Structure that extends from the apex to the medial dorsal region of the aedeagus. |
| Apical Groove (AG) | A groove between the two side lumps of the apex of the *D. lusaltans* aedeagus. |
| Anal Plates (AP) | Plate located in the cercus (a structure that belongs to the epandrium) that covers the anus. |
| Bipartite Hook (BH) | A hook located in the apex of the aedeagus of *D. emarginata.* |
| Cercus (C) | Terminalia structure formed by two annal plates. |
| Cuticular Prolongation (CP) | Prolongation linking the body to the apodeme of the aedeagus in *D. neocordata*. |
| Decasternum (D) | Structure that connects the surstyli, also known as the bridge. |
| Epandrium (E) | Tergite 9 |
| Frontal Process (FrP) | Processes that protrude from the apex to the ventral surface of the aedeagus. |
| Hypandrium (H) | A triangular structure in which the aedeagus is anchored. Sternite 9 |
| Middle Ventral Process (MVP) | Longitudinal projection present in species of the *sturtevanti* subgroup |
| Phallotreme (Ph) | A cleft located at the apex of aedeagus where the sperm is expelled |
| Serrated Plates (SP) | Lateral region of the epandrium, parallel to the cercus. |
| Sickle-Shaped Process (SSP) | Sickle-shaped ventral process of the aedeagus present in *D. neocordata.* |
| Surstylus (S) | Structure with teeth (primary and secondary) that covers the aedeagus. |
| Surstylus Process (SPr) | Process shaped like a glove finger that is located above the surstylus. |
| Scales (Sc) | Structures similar to scales found in species of the *saltans* group. |
| Teeth (T) | Structures similar to teeth that are located in the surstylus of *saltans* group species. Teeth may be primary or secondary. |
| Ventral lobe (VP) | Ventral projections of the epandrium that are extended transversely through the ventral region of the terminalia. |
| Ventral Paramere of Aedeagus (VPA) | Longitudinal structure that may or may not have spines. It is responsible for anchoring the aedeagus at the hypandrium. |
| Ventral Process (VPr) | Ventral structure that extends from the basal region to the dorsal region of the aedeagus body. |
